# Effect of Whole-Body Electromyostimulation Training on Glycemic Control in People With Prediabetes: Protocol for a Pilot Randomized Controlled Trial Study

**DOI:** 10.2196/68761

**Published:** 2025-06-24

**Authors:** Mahdieh Shojaa, Katharina Knaub, Norbert Schmitz, Andreas Michael Nieß, Barbara Munz, Sarah Rau, Viktoria Feit, Wallen Mphepo, Rahel Dingler, Wolfgang Kemmler

**Affiliations:** 1 Department of Population-Based Medicine University Hospital Tübingen Tübingen Germany; 2 Department of Sports Medicine University Hospital Tübingen Tübingen Germany; 3 Evidence-Based Healthcare Programme University of Oxford Oxfordshire United Kingdom; 4 Institute of Radiology University Hospital Erlangen Erlangen Germany

**Keywords:** prediabetes, prevention, type 2 diabetes mellitus, whole-body electromyostimulation, lifestyle intervention, activity tracker

## Abstract

**Background:**

Diabetes prevention programs focus on people with prediabetes because they have a greater risk of developing type 2 diabetes mellitus than people with normal blood glucose levels. Weight management can reduce this risk. However, in our largely sedentary society, there is less enthusiasm for regular exercise. Whole-body electromyostimulation (WB-EMS) is a training technology that provides exercise-like effects by inducing muscle contractions using electrical currents. There is evidence that local EMS can improve glucose metabolism. Several studies investigated the effect of WB-EMS on cardiometabolic risk factors including blood glucose control in a population of individuals with metabolic syndrome. However, to the best of our knowledge, there is no randomized controlled trial examining the preliminary efficacy of WB-EMS on hemoglobin A_1c_ (HbA_1c_) levels in individuals with prediabetes.

**Objective:**

The objective of this randomized controlled trial is to pilot procedures for a randomized controlled trial testing WB-EMS training on glycemic changes in sedentary adults with prediabetes.

**Methods:**

A total of 60 community-dwelling sedentary adults aged 40-65 years with prediabetes will be randomized to one of 3 arms: WB-EMS + an activity tracker and a lifestyle education program (LEP) focusing on diabetes prevention, an activity tracker and LEP, or LEP only, with 20 individuals in each arm. The WB-EMS training will consist of 1.5×20 minutes per week. The intervention will last 16 weeks. As a pilot study, our main outcomes concern the number of participants who will be recruited, and comply with intervention and follow-up. The primary efficacy outcome of interest includes hemoglobin A_1c_. The intention-to-treat analysis will be conducted with the objective of providing CI estimation of treatment effects.

**Results:**

The recruitment of study participants started in February 2024. At the time of submission of this protocol for publication, the recruitment was still ongoing. As of October 2024, a total of 42 participants were allocated to the study groups. The anticipated date of recruitment completion is July 2025.

**Conclusions:**

The results of this trial will provide valuable evidence for future investigations comparing the efficacy of the WB-EMS intervention with traditional exercise training to improve glycemic control in this population.

**Trial Registration:**

ClinicalTrials.gov NCT06188481; https://clinicaltrials.gov/study/NCT06188481

**International Registered Report Identifier (IRRID):**

DERR1-10.2196/68761

## Introduction

### Overview

Type 2 diabetes mellitus (T2DM) is a serious and widespread disease and is recognized as a major public health challenge worldwide. According to the International Diabetes Federation (IDF), the current estimate of adults aged 20-79 years living with diabetes equals 9.3% (463 million) and is projected to increase to 10.9% (700 million) by 2045 [1]. As the prevalence of T2DM increases, effective prevention measures are increasingly important. Focusing on those who are at high risk of developing T2DM is a pivotal starting point.

A state of glucose metabolism is called prediabetes when diabetes mellitus is not yet present but elevated fasting plasma glucose levels or impaired glucose tolerance are known [2]. Hemoglobin A_1c_ (HbA_1c_) levels between 5.7% and 6.4% are indicative of prediabetes. The prevalence of prediabetes in Germany is estimated to be 26% in people aged 45 to 64 years and 31% in people aged 65 to 69 years [3]. Similarly, in the United States, 21.7% of people have prediabetes according to the HbA_1c_ diagnostic criteria [4]. The clinical relevance of prediabetes is that there is a significantly increased risk of developing T2DM compared to the normoglycemic population, which in turn is associated with an increased risk of developing cardiovascular disease, kidney disease, cancer, depression, and dementia [2]. The results from the population-based Rotterdam Study suggest that up to 74% of 45-year-olds with prediabetes will develop T2DM during their lifetime [5].

The results from the KORA-F4 study (Cooperative Health Research in the Region of Augsburg) and the SHIP-TREND study (Study of Health in Pomerania) suggest that up to one-third of people with prediabetes can be classified as physically inactive [6]. Lifestyle changes, such as physical activity and weight management, are of critical importance in preventing the development and progression of T2DM [2,7].

Although, physical activity interventions have shown beneficial effects on oral glucose tolerance, fasting glucose, and HbA_1c_ levels in people with prediabetes [8], insufficient physical activity in people with T2DM and prediabetes remains an issue in these populations [9]. There are several barriers associated with participation and long-term adherence to exercise programs among physically inactive people with diabetes, including lack of time, lack of motivation, poorer (self-rated) health, risk of injury, and social and environmental barriers [10]. Therefore, physical activity programs with an attractive profile are needed to reduce sedentary behavior and increase adherence to physical activity. A potential approach to achieve this goal is an innovative training technology called whole-body electromyostimulation (WB-EMS). WB-EMS provides exercise-like effects by inducing muscle contractions using electrical currents from an external source. WB-EMS simultaneously stimulates up to 8-12 major muscle groups with up to 2800 cm^2^ of electrode area. Electrodes are placed on the gluteus muscle, thighs, lower back, upper back, latissimus dorsi, abdomen, chest, and upper arms, and each body region is stimulated with an individualized pulse intensity while individuals perform low-intensity functional exercises in a standing position [11].

In recent years, WB-EMS has found its way not only into therapeutic training but also into rapid and widespread distribution, particularly in Europe, with more than 2000 commercial WB-EMS providers serving approximately 250,000 clients in Germany alone [12]. Due to its increasing popularity, time efficiency, ease of implementation, joint-friendliness, and personalized application, WB-EMS is increasingly the subject of scientific research. In fact, several studies have demonstrated positive effects of WB-EMS on muscle hypertrophy and body composition [13,14]. In addition, high adherence to the intervention and low dropout rates were reported previously [14]. Therefore, this training technology can be considered a promising approach when aiming to increase physical activity in a population of sedentary adults.

A nonrandomized study of WB-EMS showed benefits to HbA_1c_ levels and fasting glucose in middle-aged and older men with T2DM [15]. In addition, several small studies have shown that local EMS has beneficial effects on glycemic control [16]. In contrast, a recent randomized crossover study by Holzer et al [17] found no significant short-term difference in the acute postprandial glucose response between exercise with and without WB-EMS. However, due to the small sample size (6 patients with T2DM) and short intervention duration (three 20-min sessions), the results of this study cannot be generalized [17]. Some studies further investigated the effect of WB-EMS in a population of individuals with metabolic syndrome. While prediabetes and metabolic syndrome share some common characteristics, people with metabolic syndrome have heterogeneous cardiometabolic profiles, where prediabetes or elevated blood sugar levels might be present, but do not necessarily occur. A study showed that 12 weeks of regular WB-EMS in combination with nutritional counseling had a beneficial effect on body weight and composition, and positively affected blood cholesterol levels, but not HbA_1c_ levels [18].

A recent randomized controlled study investigated the effect of WB-EMS in comparison to resistance training on cardiometabolic risk factors. The study found that there was no significant effect on variables including HbA_1c_ [19].

We intend to use WB-EMS focusing on HbA_1c_ in people with prediabetes and then examine its preliminary efficacy. Therefore, an appropriate design is needed to compare the HbA_1c_ of the intervention group (IG) with that of the comparison groups.

### Study Objectives

Our main objective is to pilot the feasibility of a randomized trial testing WB-EMS for HbA_1c_ levels in people with prediabetes. The primary objective is to provide an estimation of the preliminary efficacy of the intervention on glycemic control in a sedentary group of adults with prediabetes compared with control groups (CGs).

The secondary objectives are to assess the preliminary efficacy of:

1. WB-EMS training on the individual's biomarkers (eg, triglycerides, high-density lipoprotein, and low-density lipoprotein).
2. WB-EMS training on body composition, blood pressure, and heart rate.
3. WB-EMS on other outcomes, such as quality of life and stress.

## Methods

### Study Design and Setting

The trial is designed as a randomized controlled pilot trial in a parallel design with 3 arms. The trial will be conducted at the Department of Population-Based Medicine at the University Hospital of Tübingen, Germany.

### Study Population

The study population will be selected on the basis of the following inclusion and exclusion criteria (Textbox 1).

**Inclusion and exclusion criteria.**

**Inclusion criteria**

- Community-dwelling sedentary men and women aged 40-65 years without type 2 diabetes mellitus
- Elevated hemoglobin A_1c_ levels (5.7%-6.4%)
- Not functionally impaired (Short Physical Performance Battery ≥10)
- Signed informed consent
- Consent to use the whole-body electromyostimulation and activity tracker

**Exclusion criteria**

- High-grade arrhythmia, cardiac pacemaker carriers, heart failure, nephropathy
- Cognitive impairment
- Type 2 diabetes mellitus

### Study Procedure

**Figure 1:**
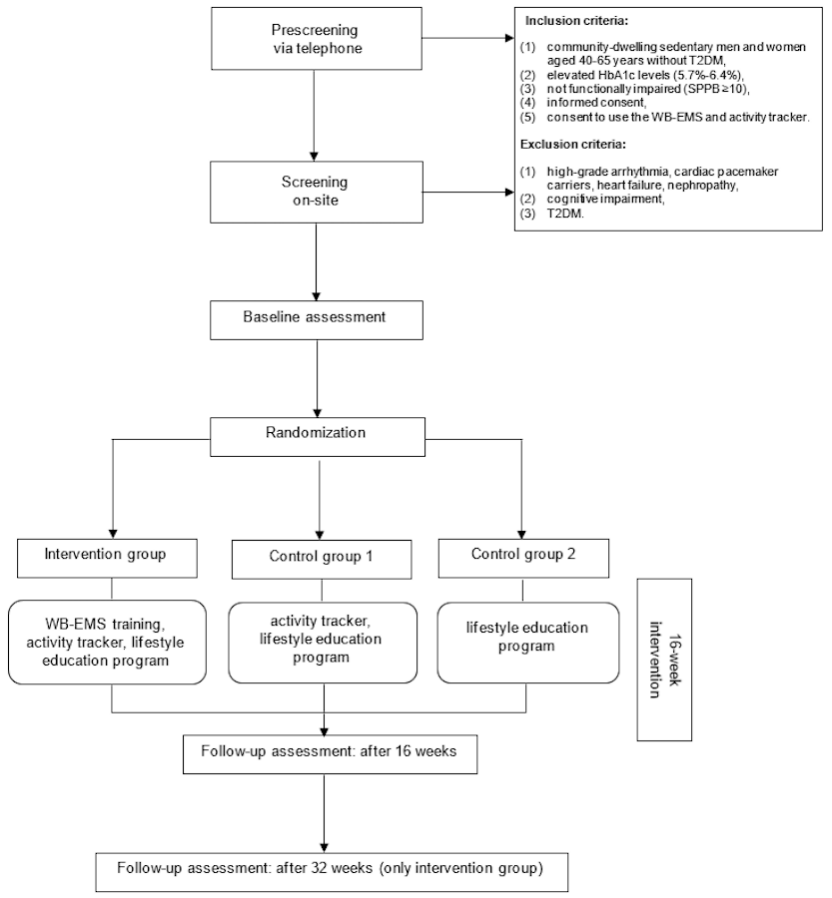
Flowchart of the study procedure. T2DM: type 2 diabetes mellitus; WB-EMS: whole-body electromyostimulation.

Figure 1 illustrates the study procedure. Screening of participants will take place in 2 stages (telephone prescreening and on-site screening). During the telephone pre-screening, the study staff will check key study eligibility criteria and inform participants about the study activities. If they are interested, they will be invited to the on-site screening. During the on-site screening, the study staff will perform the HbA_1c_ measurement. Furthermore, physical examination (eg, blood pressure) and detailed medical history will be collected to account for any possible contraindications and to ensure safe participation in the training program.

The final determination of eligibility to participate in the trial will take place after the screening. The participants will be randomized to 1 IG and 2 CGs. The duration of the intervention will be 16 weeks. At the end of the intervention period, individuals will return to the study site for a follow-up assessment. The participants of the IG will have a second follow-up assessment after 32 weeks.

### Sample Size

The study population will consist of 60 individuals with 20 individuals in each study group. As this is a pilot study, no formal power analysis has been carried out. However, the target sample size was based on recruitment for 15 months at an estimated rate of 4 patients per month (ie, 60 patients), which was considered adequate to obtain reasonably reliable sample size estimates [20-22].

### Recruitment

Participants will be recruited through newspaper and social media advertisements. Flyers and posters will also be distributed to local doctors’ offices, pharmacies, hospitals, community centers, and self-help groups.

### Randomization, Blinding, and Concealment of Allocation

Eligible participants will be allocated to the study groups by computerized block randomization. This will be performed by an independent trial statistician. Neither the participants nor the researchers will know their allocation in advance. The participants will be strictly separated after randomization. The study staff will prevent participants from different study groups from arriving at the study site at the same time by planning study visits accordingly.

The blinding strategy focuses on study assistants and outcome assessors who will be blinded to the participants’ group status. All data analysts will also be blinded to group allocation.

### Intervention

**Figure 2:**
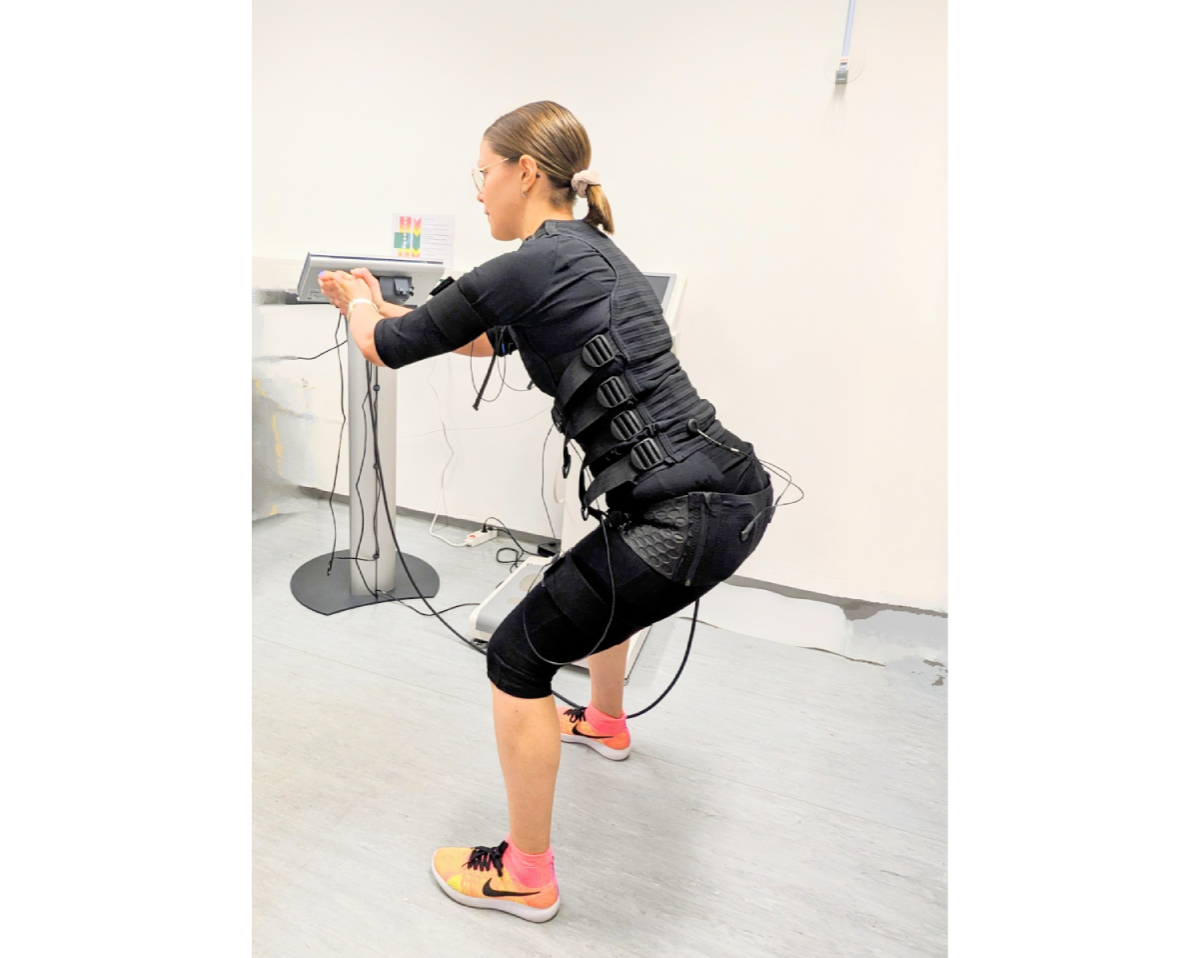
Whole-body electromyostimulation.

The main intervention will be WB-EMS using equipment that has EU (European Union) medical device approval (miha bodytec, Type II). This equipment allows the simultaneous stimulation of up to 12 muscle groups (eg, thigh and upper arm, gluteal region, abdomen, chest, lower back, and upper back) with a total stimulation area of approximately 2800 cm^2^. The participants will receive the functional training underwear recommended by the manufacturer (water-absorbent cotton or elastane mix), over which a vest (upper body) and belts (upper arms, thigh, and gluteus muscle) are individually adjusted before each session. The system allows the intensity to be adjusted for each region (Figure 2). We will use an impulse protocol and exercise setting that has been evaluated in recent studies focusing on total and regional muscle mass [13], body fat [23], and physical function [13] in older cohorts. Furthermore, we designed low-intensity exercises to minimize the effect of voluntary movements themselves as low as possible.

A bipolar electric current with a frequency of 85 Hz and an impulse width of 350 microseconds is used in an interval approach with 6 seconds of EMS stimulation with a direct impulse boost and 4 seconds of rest. During the 6-second stimulation period, low-effort movements are performed in a standing position. The intensity of the EMS will be regulated on the basis of the individual’s reported Rating of Perceived Exertion (RPE) and the Borg Category Ratio Scale (Borg-CR10) [14]. We will use RPE to generate and maintain a sufficient but tolerable intensity of EMS application. After 4 weeks of conditioning at a lower pulse intensity, participants will be encouraged to increase the intensity. The impulse intensity will be adjusted individually for each body region in close interaction with the participant. During the session, the instructors will increase the intensity slightly every 3 minutes in close cooperation with the participants in order to maintain the target RPE (6-7) during the session. We will use a personal training setting with one certified and experienced instructor responsible for a maximum of 2 participants. The training will adhere to the requirements of the international EMS guidelines [24].

### Study Groups

#### Intervention Group

The participants in the IG will train following a supervised and guided WB-EMS program 1.5 times per week (eg, every Monday or Tuesday and every other Thursday or Friday) for 16 weeks. The duration of each training session will be 20 minutes. Each session will include a 3-minute warm-up, a 15-minute main workout, and a 2-minute cool-down. In addition, participants will be asked to wear an activity tracker on their wrist for the entire 16-week study period and to participate in the lifestyle education program.

#### Control Groups

A total of 2 CGs are planned for the study. One CG will receive both the activity tracker to measure daily steps and the evidence-based lifestyle education program. The other CG will receive the lifestyle education program only.

#### Activity Tracker

The activity tracker (vivosmart 5, Garmin) will be used for self-monitoring of daily steps. Activity trackers may improve physical activity independent of the intervention (eg, people can monitor their daily steps) [25]. Therefore, it is important to include 2 CGs: one with the activity tracker to isolate the net effect of the WB-EMS intervention and one without the activity tracker.

#### Lifestyle Education Program

The evidence-based lifestyle education program consists of six 20-minute sessions that focus on lifestyle factors and diabetes prevention. The aim of the program is to provide education, information, and advice to prevent disease progression and improve quality of life and mobility. The education program includes topics such as the basics of healthy nutrition patterns, meal planning, the importance of physical activity, and stress management. Participants will receive recorded videos on each topic to ensure standardization of the content. To assess compliance, the participants will be followed up by the study staff, who will ask, whether the program was completed, or reminded to complete the program, in case of noncompliance.

#### Outcome Measures

The assessments described below will be performed at baseline (day 1) and after the 16-week intervention period. IG participants will also be assessed at 32 weeks. The timetable of the assessments is presented in Table 1. All measurements will be performed by the same research assistant, using identically calibrated equipment, in the exact same environment and at approximately the same time of day (±90 min).

**Table 1:** Table1. Timetable of study procedure and assessments.

		Study period				
		Enrollment	Allocation (day 0)	Intervention (weeks 1-16)	Follow-ups	
					Week 16	Week 32
Eligibility screening		✓				
Informed consent		✓				
Review inclusion and exclusion criteria		✓				
Allocation			✓			
**Assessments**						
	HbA_1c_^a^ assessment	✓			✓	✓
	Lipid and cholesterol profile		✓		✓	✓
	Blood pressure		✓		✓	✓
	Hand grip strength		✓		✓	✓
	Body composition		✓		✓	✓
	Waist circumference		✓		✓	✓
	Questionnaires (WHOQOL-BREF^b^, SFI^c^, PSS-10^d^, PHQ-9^e^)		✓		✓	✓
**Interventions**						
	WB-EMS^f^, activity tracker and evidence-based lifestyle education program			WB-EMS 1.5/wk Activity tracker 6 x 20 min LEP^g^		
	Activity tracker and evidence-based lifestyle education program			6 x 20 min LEP Activity tracker		
	Evidence-based lifestyle education program			6 × 20 min LEP		

^a^HbA_1c_: hemoglobin A_1c_.

^b^WHOQOL-BREF: WHO Health-Related Quality of Life.

^c^SFI: Secure Flourish Index.

^d^PSS: Perceived Stress Scale.

^e^PHQ: Patient Health Questionnaire.

^f^WB-EMS: whole-body electromyostimulation.

^g^LEP: lifestyle education program.

#### Primary Outcome

As a pilot study, our main outcome is to assess the feasibility of the proposed intervention, with specific attention to the recruitment, adherence, and completion rates of the follow-up assessments. The prespecified primary outcome measure will be HbA_1c_ levels. A blood sample (capillary blood) will be collected via the finger stick technique. There is no need for the participants to fast prior to blood measurement. The sample will be analyzed according to the manufacturer’s instructions using Cobas b 101 system 2.0 (Roche Diagnostics).

#### Secondary Outcomes

##### Cardiometabolic Parameters

Changes from baseline in the levels of triglycerides, total cholesterol as well as high-density lipoprotein and low-density lipoprotein cholesterol will be assessed in capillary blood. A blood sample will be collected with the finger stick technique. The sample will be analyzed according to the manufacturer’s instructions using Cobas b 101 system 2.0 (Roche Diagnostics).

Waist circumference will be measured with a standard tape measure around the abdomen between the distal end of the rib cage and the top of the iliac crest along the horizontal plane (World Health Organization [WHO] guidelines).

In addition, changes in systolic and diastolic blood pressure as well as in heart rate will be investigated (boso medicux X, BOSCH + SOHN). The measurements will be performed in the sitting position after a short resting period of at least 5 minutes.

Body composition: Body mass and composition will be measured by direct segmental, multifrequency bioimpedance technique (InBody 770). This device measures the impedance of the trunk, arms, and legs separately using a tetrapolar 8-point tactile electrode system that applies 6 frequencies (1, 5, 50, 250, 500, and 1000 kHz).

##### Muscle Strength

Hand grip strength will be measured using a hydraulic hand dynamometer SAEHAN SH5001 (SAEHAN Corporation). The dynamometer grip width will be adjusted individually to the participant’s hand size. Tests will be performed in an upright standing position, arms down by the side. The standardized instruction to the participants will be consistently “squeeze as strongly as possible.” Three tests intermitted by 20 seconds of rest will be performed for the dominant hand and the average value will be used for the evaluation.

##### Stress

A self-reported questionnaire Perceived Stress Scale consisting of 10 questions about stress (PSS-10) will be used [26]. In each question, individuals are asked how often they feel a certain way on a 5-point scale from 1 for “never” to 5 for “very often.” The PSS score indicates levels of perceived stress, whereby higher scores indicate higher stress levels.

##### Depressive Symptoms

The Patient Health Questionnaire which consists of 9 questions on depressive symptoms (PHQ-9) is a standardized and validated self-reported questionnaire. It will be used to assess for changes from baseline regarding the presence and severity of such symptoms. The possible score ranges from 0 (no depression) to 27 (severe depression) [27].

##### Health-Related Quality of Life

The standardized and validated questionnaire WHO Health-Related Quality of Life (WHOQOL-BREF) consisting of 26 questions on general quality of life will be used [28]. This self-reported tool includes 4 domains: physical health, psychological health, social relationships, and environmental health. It also contains items about quality of life and general health. Each item of the WHOQOL-BREF is scored from 1 to 5 on a 5-point Likert scale. The scores are then transformed linearly to a 0-100 scale. A higher score indicates a higher quality of life.

##### Well-Being

Changes from baseline in self-reported well-being will be evaluated with the standardized and validated questionnaire Secure Flourish Index (SFI), which consists of 12 questions on general well-being. Each of the questions is assessed on a scale of 0-10. The SFI score is obtained by summing the scores from the 12 questions and results in a score from 0 to 120 [29].

### Baseline Characteristics

Table 1 describes the type of data and variables that are collected at various stages of the project. Furthermore, the following demographic and descriptive data are collected at the baseline assessment: gender, age, height (cm), medical history (diseases, surgery, and medicine consumption), physical activity (type, frequency, and duration), educational level, and employment status.

Trainers will monitor adverse events by asking participants if they have experienced any health problems (eg, physical injury and muscle soreness) since the last session. The research team will monitor any adverse events that may occur during participation in this study.

### Data Management and Analysis

#### Data Collection and Storage

For the scientific analysis, the data will be stored electronically in a pseudonymized form by assigning a participant ID to each study participant. The list of participant codes and participants will be kept in a locked cabinet and will be accessible only to authorized personnel.

#### Activity Tracker Data

The tracker can be set to measure a customizable set of matrices, as needed for the project to avoid unnecessary data collection. This pilot study will collect and process step counts. The Fitrockr Health Data Research & Analytics Platform provides a platform for research and clinical trials to collect and analyze data from wearable devices. Authorized members of the research team will access the device data via Bluetooth or a USB cable using the Fitrockr Hub App when the study participants are on-site. All data will be securely stored on a server in Germany. The participants’ usernames will be generated according to the participant ID.

### Statistical Analysis

The data will be analyzed using the intention-to-treat principle, which includes all randomized participants. Descriptive statistics, such as mean and SD will be performed for continuous variables, and frequencies and percentages will be used for categorical variables. Diagnostic tests will be conducted for the normality of residuals, linearity, multicollinearity, functional relation, influence, and outliers in the dataset. Assuming data normality, 2-way repeated measures ANOVA and 95% CI will be used to assess outcomes at baseline and 4-month follow-up. The analysis plan will not include reporting of *P* values, in accordance with the CONSORT (Consolidated Standards of Reporting Trials) 2010 statement: extension to randomized pilot and feasibility trials that “any estimates of effect using participant outcomes as they are likely to be measured in the future definitive randomized controlled trial (RCT) would be reported as estimates with 95% CIs without *P* values—because pilot trials are not powered for testing hypotheses about effectiveness” [30].

Analysis of covariance will be used to calculate the effect estimate (study outcomes) of the intervention. Effect size calculation for between-group intervention effects and within-group effects will also be performed. All statistical analyses will be performed using SPSS Statistics (version 28, IBM Corp).

### Ethical Considerations

This trial protocol version 1 from August 2023 was approved by the local Ethics Committee at the Medical Faculty of the Eberhard Karls University and at the University Hospital of Tübingen (reference: 525/2023BO2) and registered on the ClinicalTrials.gov (ID NCT06188481). Following the approval of the ethics committee on November 6, 2023, preparations for the advertisement and implementation of the project, including the registration of the clinical trial on ClinicalTrials.gov (ID: NCT06188481), were conducted in early January 2024.

All participants will receive a written information letter and informed consent form for participation. All participants will be informed of the voluntary nature of the trial and that they may withdraw from the trial at any time without giving a reason. Only those who confirm their willingness to participate in the trial by signing the informed consent form will be enrolled.

The identities of all participants will be pseudonymized before starting the collection of study data to protect participant identities. The personal data of the participants will be treated confidentially and will only be accessible to the persons directly involved in the study. Further processing, analysis, and publication of the data for scientific purposes will be done anonymously so that no conclusions can be drawn about individual study participants.

Participants in both control groups will be compensated for their time with a coupon for a fitness class at the adult education center (Volkshochschule) valued at €80 (US $91.76). Additionally, all participants will receive feedback about their health parameters. The compensation will be communicated to the participants before enrollment to ensure transparency in the compensation process.

## Results

The recruitment of participants and on-site screening started at the beginning of February 2024. At the time of submission of this protocol for publication, the recruitment was still ongoing. As of October 2024, a total of 169 individuals have registered for the study. Of these, 76 individuals were invited to attend the on-site screening, and the rest were excluded during the telephone prescreening process. Finally, 47 participants were eligible and 42 of those were randomized and allocated to the study groups. The 16-week intervention phase starts in groups of at least 6 participants (block randomization) in order to ensure randomized allocation to the 3 study groups. The use of sealed opaque envelopes ensured that neither participants nor researchers were aware of the allocation prior to the study (ie, allocation concealment).

The study is still in progress, and thus far, no adverse events have been recorded. The anticipated date of recruitment completion is of July 2025.

## Discussion

While exercise is regarded as one of the key components in T2DM management and prevention [31], adherence to regular exercise still remains low in people with T2DM and prediabetes [9]. Barriers to exercise adherence include the lack of time, lack of motivation and enthusiasm, physical discomfort, and social and environmental barriers [10]. Due to the ease of implementation, time efficiency, and personalized application of WB-EMS, it is increasingly the subject of scientific research.

This randomized clinical trial is designed to evaluate the preliminary efficacy of 16 weeks of WB-EMS training on changes in HbA_1c_ levels. The results of the current study will not only give new insights into the effect of WB-EMS in prediabetes but also serve as a foundation for future studies in this population. This may make a big contribution to the development of preventive strategies specifically for type 2 diabetes.

A meta-analysis of 35 studies reported that local EMS intervention lowered fasting blood glucose (standardized mean difference 0.48, 95% CI 0.17-0.78; *P*=.002; *I*²=0%) [16]. In a recent 16-week non-RCT in the Netherlands, WB-EMS was used to reduce visceral fat in older patients with non–insulin-dependent diabetes mellitus. The exercise program consisted of 20 minutes of WB-EMS twice a week, with HbA_1c_ measured at baseline and the end of the intervention. However, the CG consisted of healthy participants without diabetes, which led to a non-HbA_1c_ comparison between groups. Only male patients showed a significant change in HbA_1c_ (mean 55.7, SD 12.7 mmol/mol vs mean 52.7, SD 13.8 mmol/mol; *P*<.05), while female patients did not experience a significant change [32]. To the best of our knowledge, there is currently no other RCT evaluating the effect of WB-EMS on glycemic control in people with prediabetes.

HbA_1c_ is considered the gold standard for long-term blood glucose monitoring as it reflects average levels over a period of 2-3 months. Studies have shown that exercise programs typically take longer than 12 weeks to impact HbA_1c_ levels, which is similar to the lifespan of a red blood cell. Most interventional exercise studies that demonstrate improved HbA_1c_ levels have a duration of approximately 13 weeks [33]. In addition, previous studies using WB-EMS have shown significant improvement in muscle mass after a 16-week intervention with similar training frequency and impulse protocol as those used in our study [13,23]. Accordingly, the WB-EMS intervention will be conducted over a period of 16 weeks in this study.

To monitor the level of physical activity, participants in the IG will be asked to wear an activity tracker during the intervention. However, there is evidence of a positive effect of activity trackers on physical activity behavior through personalized feedback on daily steps [25]. Therefore, some of the participants will be randomized to an additional CG with an activity tracker in order to isolate the net effect of the WB-EMS intervention.

The results of this study may provide evidence of the preliminary efficacy of WB-EMS for a future definitive RCT as an alternative adoptive exercise approach to motivate sedentary people with prediabetes who are unable or choose not to follow a traditional exercise program. As this is the first RCT with WB-EMS training to focus on individuals with prediabetes and to address HbA_1c_ as the primary outcome, the results of the trial will inform the sample size calculation for a definitive trial. Moreover, we believe that this research study will provide additional evidence for the health and performance aspects of the WB-EMS application.

## Abbreviations

Borg-CR10: Borg Category Ratio Scale
CG: control group
CONSORT: Consolidated Standards of Reporting Trials
EU: European Union
HbA_1c_: hemoglobin A_1c_
IDF: International Diabetes Federation
IG: intervention group
LEP: lifestyle education program
PHQ-9: Patient Health Questionnaire
PSS: Perceived Stress Scale
RCT: randomized controlled trial
RPE: Rating of Perceived Exertion
SFI: Secure Flourish Index
T2DM: type 2 diabetes mellitus
WB-EMS: whole-body electromyostimulation
WHO: World Health Organization
WHOQOL-BREF: World Health Organization Health-Related Quality of Life

## References

[1] International Diabetes Federation (2019). IDF Diabetes Atlas. 9th Edition.

[2] American Diabetes Association (2022). Abridged for Primary Care Providers. Clin Diabetes.

[3] Heidemann C, Du Y, Paprott R, Haftenberger M, Rathmann W, Scheidt-Nave C (2016). Temporal changes in the prevalence of diagnosed diabetes, undiagnosed diabetes and prediabetes: findings from the German Health Interview and Examination Surveys in 1997-1999 and 2008-2011. Diabet Med.

[4] Herman WH (2023). Prediabetes diagnosis and management. JAMA.

[5] Ligthart S, van Herpt TTW, Leening MJG, Kavousi M, Hofman A, Stricker BHC, van Hoek M, Sijbrands EJG, Franco OH, Dehghan A (2016). Lifetime risk of developing impaired glucose metabolism and eventual progression from prediabetes to type 2 diabetes: a prospective cohort study. Lancet Diabetes Endocrinol.

[6] Stöckl D, Rückert-Eheberg I, Heier M, Peters A, Schipf S, Krabbe C, Völzke H, Tamayo T, Rathmann W, Meisinger C (2016). Regional variability of lifestyle factors and hypertension with prediabetes and newly diagnosed type 2 diabetes mellitus: the population-based KORA-F4 and SHIP-TREND studies in Germany. PLoS ONE.

[7] Campbell TJ, Alberga A, Rosella LC (2016). The impact of access to health services on prediabetes awareness: a population-based study. Prev Med.

[8] Rossen J, Von Rosen P, Johansson U, Brismar K, Hagströmer M (2019). Associations of physical activity and sedentary behavior with cardiometabolic biomarkers in prediabetes and type 2 diabetes: a compositional data analysis. Phys Sportsmed.

[9] Mortensen SR, Skou ST, Brønd JC, Ried-Larsen M, Petersen TL, Jørgensen LB, Jepsen R, Tang LH, Bruun-Rasmussen NE, Grøntved A (2023). Detailed descriptions of physical activity patterns among individuals with diabetes and prediabetes: the Lolland-Falster Health Study. BMJ Open Diabetes Res Care.

[10] Thielen SC, Reusch JEB, Regensteiner JG (2023). A narrative review of exercise participation among adults with prediabetes or type 2 diabetes: barriers and solutions. Front Clin Diabetes Healthc.

[11] Kemmler W, von Stengel S, Kohl M, Rohleder N, Bertsch T, Sieber CC, Freiberger E, Kob R (2020). Safety of a combined WB-EMS and high-protein diet intervention in Sarcopenic obese elderly men. Clin Interv Aging.

[12] Kemmler W, Kleinöder H, Fröhlich M (2020). Editorial: Whole-body electromyostimulation: a training technology to improve health and performance in humans?. Front Physiol.

[13] Kemmler W, Grimm A, Bebenek M, Kohl M, von Stengel S (2018). Effects of combined whole-body electromyostimulation and protein supplementation on local and overall muscle/fat distribution in older men with Sarcopenic obesity: the randomized controlled Franconia Sarcopenic Obesity (FranSO) Study. Calcif Tissue Int.

[14] Kemmler W, Weissenfels A, Teschler M, Willert S, Bebenek M, Shojaa M, Kohl M, Freiberger E, Sieber C, von Stengel S (2017). Whole-body electromyostimulation and protein supplementation favorably affect sarcopenic obesity in community-dwelling older men at risk: the randomized controlled FranSO study. Clin Interv Aging.

[15] van Buuren F, Horstkotte D, Mellwig KP, Fründ A, Vlachojannis M, Bogunovic N, Dimitriadis Z, Vortherms J, Humphrey R, Niebauer J (2015). Electrical myostimulation (EMS) improves glucose metabolism and oxygen uptake in type 2 diabetes mellitus patients--results from the EMS study. Diabetes Technol Ther.

[16] Sanchez MJ, Mossayebi A, Sigaroodi S, Apaflo JN, Galvan MJ, Min K, Agullo FJ, Wagler A, Bajpeyi S (2023). Effects of neuromuscular electrical stimulation on glycemic control: a systematic review and meta-analysis. Front Endocrinol.

[17] Holzer R, Schulte-Körne B, Seidler J, Predel H, Brinkmann C (2021). Effects of acute resistance exercise with and without whole-body electromyostimulation and endurance exercise on the postprandial glucose regulation in patients with type 2 diabetes mellitus: a randomized crossover study. Nutrients.

[18] Reljic D, Konturek PC, Herrmann HJ, Neurath MF, Zopf Y (2020). Effects of whole-body electromyostimulation exercise and caloric restriction on cardiometabolic risk profile and muscle strength in obese women with the metabolic syndrome: a pilot study. J Physiol Pharmacol.

[19] Reljic D, Herrmann HJ, Neurath MF, Zopf Y (2021). Iron beats electricity: resistance training but not whole-body electromyostimulation improves cardiometabolic health in obese metabolic syndrome patients during caloric restriction-a randomized-controlled study. Nutrients.

[20] Kunselman AR (2024). A brief overview of pilot studies and their sample size justification. Fertil Steril.

[21] Julious SA (2005). Sample size of 12 per group rule of thumb for a pilot study. Pharmaceut Statist.

[22] Van BG (2008). Statistical Rules of Thumb, 2nd Edition.

[23] Kemmler W, Teschler M, Weißenfels A, Bebenek M, Fröhlich M, Kohl M, von Stengel S (2016). Effects of whole-body electromyostimulation versus high-intensity resistance exercise on body composition and strength: a randomized controlled study. Evid Based Complement Alternat Med.

[24] Kemmler W, Fröhlich M, Ludwig O, Eifler C, von Stengel S, Willert S, Teschler M, Weissenfels A, Kleinöder H, Micke F, Wirtz N, Zinner C, Filipovic A, Wegener B, Berger J, Evangelista A, D'Ottavio S, Sara JDS, Lerman A, Perez de Arrilucea Le Floc'h UA, Carle-Calo A, Guitierrez A, Amaro-Gahete FJ (2023). Position statement and updated international guideline for safe and effective whole-body electromyostimulation training-the need for common sense in WB-EMS application. Front Physiol.

[25] Ellingson LD, Lansing JE, DeShaw KJ, Peyer KL, Bai Y, Perez M, Phillips LA, Welk GJ (2019). Evaluating motivational interviewing and habit formation to enhance the effect of activity trackers on healthy adults' activity levels: randomized intervention. JMIR Mhealth Uhealth.

[26] Cohen S, Williamson G, Spacapan S, Oskamp S (1988). Perceived stress in a probability sample of the United States. The Social Psychology of Health: Claremont Symposium on Applied Social Psychology.

[27] Kroenke K, Spitzer RL, Williams JBW (2001). The PHQ-9: validity of a brief depression severity measure. J Gen Intern Med.

[28] Kim S, Michalos A.C. (2014). World Health Organization Quality of Life (WHOQOL) Assessment. Encyclopedia of Quality of Life and Well-Being Research.

[29] VanderWeele TJ (2017). On the promotion of human flourishing. Proc Natl Acad Sci U S A.

[30] Eldridge SM, Chan CL, Campbell MJ, Bond CM, Hopewell S, Thabane L, Lancaster GA, PAFS consensus group (2016). CONSORT 2010 statement: extension to randomised pilot and feasibility trials. BMJ.

[31] Kanaley JA, Colberg SR, Corcoran MH, Malin SK, Rodriguez NR, Crespo CJ, Kirwan JP, Zierath JR (2022). Exercise/physical activity in individuals with type 2 diabetes: a consensus statement from the American College of Sports Medicine. Med Sci Sports Exerc.

[32] Houdijk APJ, Bos NFJME, Verduin WM, Hijdendaal MM, Zwartkruis MAL (2022). Visceral fat loss by whole-body electromyostimulation is attenuated in male and absent in female older non-insulin-dependent diabetes patients. Endocrinol Diabetes Metab.

[33] Grace A, Chan E, Giallauria F, Graham PL, Smart NA (2017). Clinical outcomes and glycaemic responses to different aerobic exercise training intensities in type II diabetes: a systematic review and meta-analysis. Cardiovasc Diabetol.

